# Cerebral palsy in children in Kampala, Uganda: clinical subtypes, motor function and co-morbidities

**DOI:** 10.1186/s13104-015-1125-9

**Published:** 2015-04-23

**Authors:** Angelina Kakooza-Mwesige, Hans Forssberg, Ann-Christin Eliasson, James K Tumwine

**Affiliations:** Department of Paediatrics & Child Health, Makerere University College of Health Sciences, P O Box 7072, Kampala, Uganda; Department of Women’s & Children’s Health, Astrid Lindgren Children’s Hospital, Neuropediatric Research Unit, Karolinska Institutet, Stockholm, Sweden

**Keywords:** Birth asphyxia, Cerebral palsy, Children, Co-morbidity, Motor function, Emergency obstetric care, Uganda

## Abstract

**Background:**

Cerebral palsy (CP) is a common chronic childhood disorder worldwide. There is limited information about the CP panorama in sub-Saharan Africa. Our aim was to describe the clinical subtypes, gross and fine motor functions and presence of co-morbidities in a group of children with CP attending a tertiary hospital in Uganda.

**Methods:**

Children with CP in the age range of 2-12 years visiting the paediatric CP clinic at Mulago Hospital, Kampala, were enrolled. Screening and inclusion were based on a three-stage procedure: i) Two screening questions from the Ten Question Screen; ii) Clinical assessments adapted from the Surveillance for Cerebral Palsy in Europe (SCPE); iii) Clinical examinations and diagnoses of subtype, severity level and co-morbidities. Caregivers were interviewed using questionnaires to provide information on child’s medical history and co-morbidities. Co-morbidity scores were calculated for each child.

**Results:**

One hundred and thirty five children with CP were enrolled (72 males, 63 females, median age 3 years 5 months, IQR-2 years 4 months-5 years 6 months). Bilateral spastic type was commonest (45%); moderate impairment in gross motor function was present in 43%, with comparable numbers (37%) in the mild and severely impaired fine motor function groups. The severe gross and fine motor function levels were seen in the bilateral spastic and dyskinetic CP subtypes.

Signs of learning disability (75%) and epilepsy (45%) were the commonest co-morbidities. Higher co-morbidity scores were obtained in children with dyskinetic CP and severe levels of gross and fine motor function. There was a significant difference in distribution of the co-morbidity scores between the CP subtypes, gross motor and fine motor function levels (p <0.001). Signs of speech and language impairments were associated with bilateral spastic CP and severe gross and fine motor dysfunction (p < 0.05).

**Conclusions:**

Bilateral spastic CP was the main clinical subtype, with signs of learning disability and epilepsy as major causes of co-morbidity. The severity of gross and fine motor function levels was related to severity of clinical CP subtypes. Our findings imply a higher occurrence of birth asphyxia or post natally acquired infections. Improvement in emergency obstetric and postnatal care may reduce this burden.

**Electronic supplementary material:**

The online version of this article (doi:10.1186/s13104-015-1125-9) contains supplementary material, which is available to authorized users.

## Background

Cerebral palsy (CP) is a neuro-developmental condition comprising a group of permanent disorders of movement and posture that are attributed to non-progressive disturbances of the developing foetal/infant brain. Prevalence estimates from High Income Countries (HIC) [1,2] range from 2.2-3.3/1,000 while those in Africa are less precise with limited information depicting wide ranging figures from 2-10/1,000 live births [3,4]. The variation may depend on study populations, locations, data sources, diagnostic criteria and the time periods in which the studies were done. There may however be true differences between sub-Saharan African (SSA) countries and other regions depending on the living conditions, maternal and child health care practices, delivery support, infections and a diverse disease burden.

Despite lack of reliable information on the prevalence of CP in Uganda, there is reason to believe that this condition is significant in view that for every 1,000 live births in Uganda approximately five children (5.4) do not live to their first birthday and four women (4.38) die during pregnancy and its related complications [5]. This is magnified by the frequent exposures to virus and other infections during pregnancy, greater birth trauma, malnutrition, HIV/AIDS and complications of cerebral infections especially cerebral malaria and meningoencephalitis.

The Surveillance of Cerebral Palsy in Europe (SCPE) classification is one of the international clinical classification schemes for CP where by diagnosis of the clinical subtype is based on the dominant type of movement disorder and/or distribution pattern of trunk or limb involvement, whether spastic, ataxic or dyskinetic [6]. Although the *sine qua non* for CP syndromes is impaired motor function, 25-80% have additional non-motor impairments including: disturbances of sensation, perception, cognition, communication and behaviour as well as epilepsy and other medical disorders [7].

Whereas there is ample information on the type, level of severity of motor symptoms and co-morbidities of CP in HIC, [8] there are limited studies in Low and Middle Income countries (LMIC) especially in Africa [9,10]. In order to get information about the situation in a sub-Saharan region we studied children with CP in Mulago Hospital, Kampala. The aim of this study was to describe and analyse the clinical subtype, gross and fine motor function and presence of co-morbidities in children with CP in Uganda.

## Methods

### Participants and assessments

**Figure 1:**
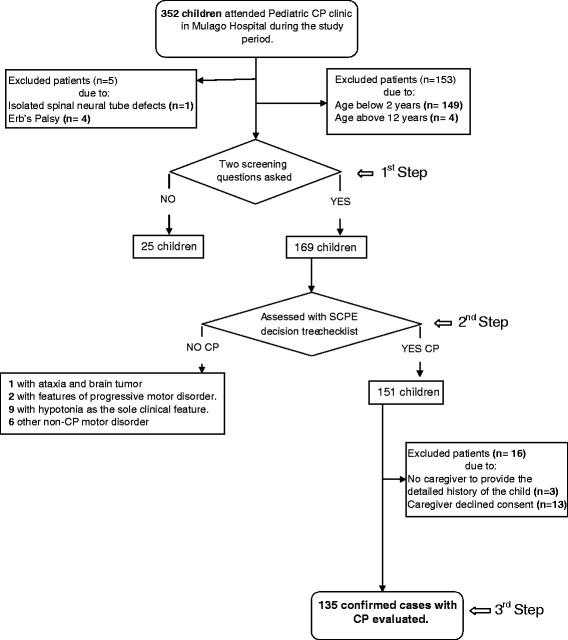
Flow chart showing sample selection and recruitment of the CP children.

We conducted a cross sectional hospital based study at Mulago, Uganda’s national referral and teaching hospital, whose catchment area encompasses the whole country and parts of the neighbouring countries. From September 2009 to August 2010 all children at the paediatric CP clinic between 2 to 12 years were identified. This clinic has a turnover of about 400 children annually with many self-referrals having different neurological symptoms. First the screening was done in two steps by a specially trained physiotherapist. Children were thereafter examined by a medical doctor and the principal investigator (AKM, trained in paediatric neurology). All consecutive cases who qualified for the inclusion criteria were recruited within one year. A summary of recruitment and inclusion in the study is shown in Figure 1.

### First step- screening

Children were screened by the physiotherapist using two questions from the Ten Question Screen (Questions 1 and 5) that correlate with motor disability [11] namely:

*1. Compared with other children, did the child have any serious delay in sitting, standing or walking?*

*2. Does the child have difficulty in walking or moving his/her arms or does he/she have weakness and/or stiffness in the arms or legs?*

These questions were translated into the local language (*Luganda*) and back translated by a team of certified professional translators and administered to caregivers in either English or *Luganda*.

### Second step -inclusion

Those that screened positive on the two questions underwent further assessments by the physiotherapist, assisted by an observer flow chart, adapted from the ‘DECISION TREE for identifying CP’ from the SCPE [6]. The SCPE diagnoses CP from the age of 4 years and above. We however undertook special consideration with ascertainment of cases made only when child was beyond age of 2 years, with no reassessments made at 4 years of age. Children with progressive motor disorders, hypotonia as the sole clinical feature or isolated spinal neural tube defects were excluded.

### Third step- assessment

If the definition of CP and other inclusion criteria were fulfilled, eligible children were confirmed by the principal investigator (AKM). Diagnostic criteria was: (a) bilateral spastic; (b) unilateral spastic; (c) dyskinetic and (d) ataxic [6] (See Additional file 1). In addition, sixteen children were excluded because either the caregivers were unable to provide the history of the child or declined consent (See Figure 1).

The severity of motor impairment involving the children’s gross and fine motor function was graded into three categories: mild, moderate and severe based on the child’s ability to sit, self-initiated walking and grasp and fine motor skills (See Additional file 2). A question regarding the acquisition of finger feeding (See Additional file 3: Appendix S1) was used as a proxy to measure level of early fine motor development [12].

The medical doctor and the physiotherapist used a pretested and pre-coded questionnaire to interview the caregiver. It included questions related to the child’s medical history from pregnancy to the present, family history, and the child’s development and nutrition.

Poor maternal nutrition was gleaned from the history provided by the caregiver indicating minimal or hardly any consumption of the nutritious foods recommended during pregnancy.

Information on co-morbidities was specifically sought during the caregiver interview and during the clinical examination. When available, a review of medical records was made. Presence of microcephaly and hydrocephalus was noted [13-15]. Signs of co-morbidities investigated included: *epilepsy*, defined when there were two or more afebrile seizures reported in the last 5 years that were spaced 24 hours apart and were unrelated to acute infection, metabolic disturbance, or drugs [16]. From the presenting symptoms and signs gathered from history and neurological examination respectively, a child was classified as having *autism spectrum disorders*, identified using the DSMIV-TR criteria [17]; *cognitive/intellectual and behavioural disorders* using the ICD-10 classification of Mental and Behavioural disorders [18]. We also included *visual impairments* which, for children less than 3 years, was assessed by observation for eye contact, and the ability to follow torch light or bright object, while a picture chart was used for those above 3 years [19]. *Hearing impairment* was garnered from the history and clinical judgment and further checked to determine whether it was severe or profound by referral for audiometric testing. *Speech and language impairment* was considered for those who were nonverbal, having the absence of specific words as well as their comprehension of words in the child’s maternal language irrespective of the possible aetiology.

### HIV status

All children were screened using the ABOTT DETERMINE® test. For those with a positive ABOTT DETERMINE® test confirmation of the child’s serostatus was done using STATPACK® test. If STATPACK® was negative the UNIGOLD® test was used as a tie-breaker. Post-test counselling was done for all patients prior to receipt of the test results.

### Ethical approval

Ethical clearance was granted by the School of Medicine Research and Ethics Committee, Makerere University College of Health Sciences and the Uganda National Council for Sciences and Technology (Reference HS 628). Caregiver informed written consent was obtained and assent was acquired from the children (aged 8 years and above) capable of making informed and voluntary decisions.

### Data analysis

Descriptive characteristics of the group of children with their distribution according to CP clinical subtype, and level of fine and gross motor function were established. The distribution of the co-morbidities was compared amongst the CP clinical subtypes as well as the levels of gross and fine motor function.

The total number of co-morbidities for each child was calculated to obtain a co-morbidity score. Statistical comparisons between the co-morbidity score tabulated across CP subtypes and levels of gross and fine motor function were performed using the Kruskal-Wallis H test (for non-parametric variables) to evaluate differences in medians among the groups. This was followed by post-hoc analysis to conduct pairwise comparisons amongst the different ranked dependent groups using the Mann-Whitney *U* test while protecting for Type I Error, by adjusting the *a priori* alpha level divided by the number of comparisons (the Bonferroni approach) [20].

All *p* values were two sided with a probability level of *p < 0.05* considered statistically significant. Data analysis was performed using SPSS Statistics software version 17.0 (SPSS Inc., Chicago, IL, USA).

## Results

### Description of the participants

Background information and characteristics of the 135 children with CP are shown in Table 1. More than two thirds 94(69.6%) of the children were five years of age and below. The two HIV positive children (female 2 years 3 months and male 4 years 8 months) had bilateral spastic CP and were antiretroviral therapy naive. Both mothers had attended antenatal care, delivered in hospital and reported history of prolonged labour > 24 hours.

**Table 1:** **Demographic and Clinical Characteristics of Children with Cerebral Palsy (n = 135)**

	**n (%) or median (IQR)**
**Characteristic**	
Sex M/F	72/63
Age at presentation(years)	3.5 (3.2)
Birth weight (Kg) n = 113	3.2 (0.94;0.8-6.0)
Birth in Hospital	101 (74.8%)
**Residence (Urban/Rural)**	95/40
**Parental factors**	
Age of father at child’s birth (years) n = 121	30 (7.9;18-68)
Age of mother at child’s birth(years) n = 130	24 (5.4;14-45)
Mother attended antenatal care	122 (90.4%)
History of infection/fever during 1^st^ trimester	58 (43.0%)
Poor maternal nutrition during pregnancy	35 (25.9%)
**Perinatal factors** ^a^	
Vaginal delivery	103 (76.3%)
Preterm birth (<37wks GA)	18 (13.3%)
Prenatal complications	37 (27.2%)
Neonatal complications	28 (20.6%)
Post neonatal complications	25 (18.4%)
**Clinical Presentation**	
Dysmorphic features	43 (31.9%)
Microcephaly^b^	32 (33.7%)
Congenital Anomaly	4 (3.0%)
Hydrocephalous	4 (3.0%)
Positive HIV Serology^c^	2 (1.7%)
**Type of Cerebral Palsy**	
Bilateral Spastic	62 (45.9%)
Unilateral Spastic	32 (23.7%)
Dyskinetic	17 (12.6%)
Ataxia	13 (9.6%)
Unclassifiable	11(8.1%)
**Co-morbidity**	
Signs of Epilepsy	61(45.2%)
Signs of Speech and Language disorders	50 (37.0%)
Signs of Visual Impairments	40 (29.6%)
Signs of Hearing Impairments	21 (15.6%)
Signs of Behavioral disorders	
▪Signs of Anxiety/Depression	26 (19.3%)
▪Signs of Attention Deficit/Hyperactivity	46 (34.1%)
Signs of Learning disability	102 (75.6%)
Signs of Autistic Spectrum Disorders	32 (23.7%)

M-Male; F-Female; GA-Gestational age.

^**a**^ **Prenatal factors:** Prenatal period: From pregnancy until birth.; Neonatal period: From birth up to day 28.

Post neonatal period: From day 29 up to 2 years.

^**b**^ **Microcephaly**- done in 95 children < 5 years of age. ^**c**^ **Positive HIV Serology** - only 114 caregivers consented to this test.

**Figure 2:**
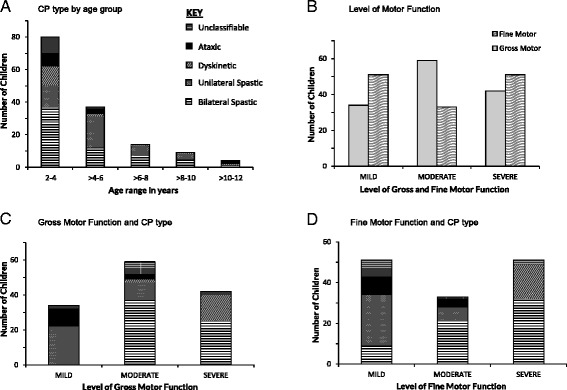
Distribution of age, clinical subtype and motor function in the CP children. Distribution of **A)** Clinical subtypes of CP by age; **B)** Levels of Fine and Gross Motor function; **C)** Clinical subtypes of CP by Gross Motor Function **D)** Clinical subtypes of CP by Fine Motor Function.

The distribution of clinical subtypes and level of gross and fine motor function are shown in Figure 2. The age group of 2-4 years had the largest mixture of all the clinical types. (Figure 2A). The majority of children were moderately impaired in gross motor function while there were similar numbers of children with mild and severely impaired fine motor function (Figure 2B).

The development of finger feeding is depicted in Additional file 4. All children with dyskinetic CP and almost half of those with bilateral spastic CP had not developed this function. Noteworthy, twenty children who had developed finger feeding function had at the time of interview moderate to severe level of fine motor function, indicating a later onset of the motor symptoms. Analysis revealed that these children reported a history highly suggestive of a postnatal brain insult such as: repeated seizures (n = 6), cerebral malaria (n = 3), febrile convulsions (n = 5) and head trauma (n = 4).

### Co-morbidities

The frequency of signs of co-morbidities is shown in Table 1. The distribution of co-morbidities in relation to clinical subtype, gross and fine motor function level is shown in Table 2. The overall most frequent co-morbidity was signs of learning disability (75.6%). Signs of attention deficit/hyperactivity 46 (34.1%) was the most common among the behavioural disorders. Most of the children had several co-morbidities. Signs of learning disability were also the most frequent co-morbidity in all clinical subtypes except for the unilateral spastic subtype in which epilepsy dominated (Table 2). When investigating the occurrence of co-morbidities in relation to gross motor function, it was noted, that the most frequent co-morbidities were found in children with moderate and severe levels of gross motor function compared to those with mild involvement.

**Table 2:** **Frequency of Co-morbidity signs in Children with Cerebral Palsy distributed by Clinical subtype and level of motor function (n = 135)**

**Parameter**	**Signs of Epilepsy**	**Signs of Speech & Language**	**Signs of Visual Disorder**	**Signs of Hearing Disorder**	**Signs of Behaviour Disorder**		**Signs of Learning Disability**	**Signs of Autism Spectrum Disorder**
					**Signs Anx/Dep**	**Signs ADHD**		
	**n = 61(%)**	**n = 50(%)**	**n = 40(%)**	**n = 21(%)**	**n = 26(%)**	**n = 46(%)**	**n= 102(%)**	**n = 32(%)**
**Clinical Subtype**								
Bilateral spastic. **N = 62**	30(49.2)	32(64.0)	24(60.0)	11(52.4)	10(38.5)	22(47.8)	51(50.0)	12(37.5)
Unilateral spastic. **N = 32**	16(26.2)	2(4.0)	5(12.5)	------	3(11.5)	11(23.9)	13(12.7)	6(18.8)
Dyskinetic. **N = 17**	10(16.4)	11(22.0)	7(17.5)	5(23.8)	7(26.9)	5(10.9)	17(16.7)	5(15.6)
Ataxic. **N = 13**	2(3.3)	2(4.0)	3(7.5)	1(4.8)	4(15.4)	6(13.0)	10(9.8)	4(12.5)
Unclassifiable. **N = 11**	3(4.9)	3(6.0)	1(2.5)	4(19.0)	2(7.7)	2(4.3)	11(10.8)	5(15.6)
**Gross Motor Function**								
Mild. **n = 34**	11(18.0)	4(8.0)	5(12.5)	2(9.5)	5(19.2)	12(26.1)	18(17.6)	8(25.0)
Moderate. **n = 59**	26(42.6)	23(46.0)	17(42.5)	10(47.6)	13(50.0)	22(47.8)	46(45.1)	14(43.8)
Severe **n = 42**	24(39.3)	23(46.0)	18(45.0)	9(42.9)	8(30.8)	12(26.1)	38(37.3)	10(31.3)
**Fine Motor Function**								
Mild. **n = 51**	17(27.9)	6(12.0)	6(15.0)	5(23.8)	10(38.5)	18(39.1)	34(33.3)	13(40.6)
Moderate. **n = 33**	11(18.0)	12(24.0)	8(20.0)	4(19.0)	8(30.8)	14(30.4)	18(17.6)	4(12.5)
Severe. **n = 51**	33(54.1)	32(64.0)	26(85.0)	12(57.1)	8(30.8)	14(30.4)	50(49.0)	15(46.9)

ADHD- Attention Deficit Hyperactivity Disorder.

Values are n (%). Each child may be counted in more than one category, and the percentages may add up to more than 100%.

Regarding fine motor function the most frequent co-morbidities were noted in children with severe impairment with the exception of the behavioural disorders that were more common in those with mild impairment.

**Figure 3:**
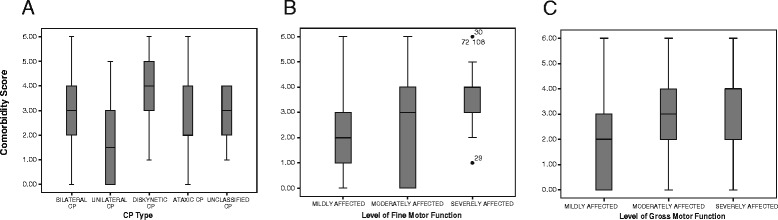
Distribution of median co-morbidity scores by clinical subtype and motor function in the CP children. Box plots showing median co-morbidity scores: box, 25 to 75%, and whisker, by **A)** Clinical type of CP; **B)** Level of Fine Motor Classification; **C)** Level of Gross Motor Function Classification.

When using co-morbidity scores, the relation to the type of CP and severity of motor functions are shown in Figure 3. The dyskinetic type of CP had the highest score and the unilateral CP the lowest. For both gross and fine motor function, the co-morbidity score increased with severity. The Kruskal-Wallis test showed that there was a significant difference in distribution of the co-morbidity scores between the CP clinical types: (X^2^ (4) = 21.51, p <0.001), the gross motor function levels (X^2^ (2) = 14.98, p = 0.001) and the fine motor function levels (X^2^ (2) = 25.60, p <0.001).

Follow-up analysis conducted to evaluate pairwise differences among the respective groups with respect to co-morbidity scores, indicated significant differences between the bilateral spastic CP and the unilateral spastic CP groups *(z = -3.527, p* <0.001); the unilateral spastic CP and the dyskinetic CP groups *(z = -3.915, p* <0.001); the mild gross motor level with the moderate and severe gross motor level groups *(z = -2.755, p* = 0.006) and *(z = -3.764, p* <0.001) respectively and the severe fine motor level with the mild and moderate fine motor level groups *(z = -5.157, p* <0.001) and *(z = -3.022, p* = 0.003) respectively.

The association between co-morbidities and type of CP (Bilateral vs other types) and level of gross and fine motor function is shown in Additional file 5. Signs of speech and language impairments were the only co-morbidity that was independently associated with bilateral spastic CP and the severe levels of gross and fine motor function.

## Discussion

The aim of this study was to begin to fill in the knowledge gap concerning the clinical panorama of CP in sub-Saharan Africa. Clinical subtypes, level of gross and fine motor function and co-morbidities were described in children visiting a CP clinic in a tertiary hospital in Uganda during one year. Bilateral spastic CP was the commonest type having a fairly even distribution across the moderate and severe levels of motor function. Signs of learning disability and epilepsy were the most frequent co-morbidities. Higher co-morbidity scores were observed for children in the subtype of dyskinetic CP and in children with severely impaired gross and fine motor function.

While there is no consensus on which is the best classification system to use to classify CP, in this study we used the SCPE classification since it has been widely adopted and reliably applied by clinicians for use in registers to describe groups, compare populations and evaluate changes in cases of intervention [6]. The finding of higher numbers (45.9%) of children with the severe form (spastic quadriplegia) of bilateral spastic CP is similar to previous clinical based studies in Africa, [9,10,21], other LMIC [22] and a 10-year case series done in Canada [23]. This pattern however differs from other hospital based studies done in LMIC’s like Turkey [8] and Bangladesh [2] that reported relatively larger numbers of the milder form (spastic diplegia) of bilateral spastic CP. The differing distribution may reflect different aetiologies. In the African studies, the larger numbers of severe bilateral spastic type point towards complications during the birth process such as birth asphyxia or towards acquired central nervous system infections such as meningitis or encephalitis [9,21] while lower frequencies of milder levels of bilateral spastic type (spastic diplegia) may reflect less recruitment from children born preterm because these children do not survive the neonatal period due to complications during the first neonatal weeks and limited support from the maternal/newborn health care service provided [24]. The probability of a Ugandan child dying within the first month of life is 27 per 1,000 live births [5]. Neonatal deaths contribute to more than one quarter of under-5 deaths in Africa with the three main causes infections, intrapartum-related conditions (“birth asphyxia”) and preterm birth together accounting for 88% of the neonatal deaths [25]. The greatest risk of death is low birth weight (<2,500 grams) including preterm birth [26], which would hence result in fewer preterm children and smaller proportion of CP recruited from this group.

Another explanation could be the differences in accessibility to tertiary health care with those with more severe disability in LMIC more likely to access care more often or be referred than those with milder forms. The parents/caregivers of children who are less severely affected may not consider their child’s problem worth referring to hospital for further management. Finally, differences in the health care might also contribute. The health care systems in the LMICs often face challenges in identification and management of these children due to many reasons ranging from: health worker and caregiver awareness and attitudes; lack of health worker training with unclear guidelines of how and where to refer [27] and lack of specialists in, e.g., paediatrics, neurology and psychiatry. This implies that most patients often have to travel long distances to seek scarce expert care and this is limited to only those able to afford the travel and consultation costs and not many of the public can afford this care. There is hence the need to advocate for the training and sustainability of multidisciplinary health disciplines at the primary health care level to meet this challenge.

Three quarters of the children were born in hospital, which is unusual given the fact that there are more Ugandan women residing in the rural areas who often use traditional birth attendants rather than the health facilities [24]. One may postulate that these mothers came to deliver in hospital when the labour was already complicated and while they might have benefited from a Caesarean section, over 75% were delivered normally. This process possibly increased the risk of birth injuries/trauma/asphyxia and may have contributed to a higher rate of CP. This postulation is supported by the finding that many Ugandan hospitals lack emergency obstetric care supplies [28] to cater for these mothers and children, yet this has been shown to be fundamental in improving the health outcome of the children [29].

Peri-natally acquired infections account for the dominant mode of acquisition of HIV infection in children [30]. For the period 2010-2011 the HIV prevalence ranged from 7.4-7.5% among Ugandan children [31]. This is in marked contrast to our finding of 1.7%. While there is no comparable information on the rate of HIV among CP children in the literature, this may be explained by selection bias or be a true reflection of the situation. Both the children with HIV in this study were born when there were limited facilities for application of the WHO (World Health Organization) and UNAIDS (The Joint United Nations Programme on HIV/AIDS) guidelines by 2007 [32] recommending routine HIV testing in all patients during clinical encounters [33]. Possibly the survival of the HIV-infected children beyond a few years of birth was very poor due to late initiation of highly active antiretroviral therapy (HAART) as in other sub-Saharan settings [34]. This suggests the possibility of earlier death of CP children with perinatally acquired HIV infection. Furthermore it emphasizes the importance of early initiation of HAART and the need to carry out additional studies to evaluate the outcome of CP in HIV-infected children.

Self-finger feeding typically develops in the latter half of the first year of the child’s life and parallels hand skill function [12]. Interestingly, 20 children with typical development of finger feeding function had lost this skill at the time of interview. Several of these had a history suggestive of a post-natal brain insult such as Cerebral Malaria and repeated seizures probably due to Meningitis. This provides a strong public health mandate to institute measures that focus on the prevention of these conditions to reduce the risk of developing post-natal CP.

Our data clearly demonstrates a relationship between the severity of motor function levels and clinical CP subtype. Severe gross and fine motor function levels were associated with bilateral spastic and dyskinetic CP while the mild motor function levels were associated with unilateral spastic CP. This is in accordance with previous studies from HIC [8], although comparison should be done cautiously since the scales [35] that are used are different.

Co morbidities in CP affect the overall health and quality of life of the individual by determining their participation in more aspects of life [36]. In our study children with signs of learning disability were most frequent followed by epilepsy. These results differ from previous studies in other parts of Africa, [9,10] which have reported epilepsy or speech and language disorders as the most frequent. Whereas it is difficult to draw parallels, the differences could partly be explained by the variability in populations in what constituted the predominant group from which the samples of the CP children were drawn, and the different methods used as well as the severity of impairment. Most children in our study were term babies and severely impaired children and this degree of impairment correlates and confirms what is known in the literature of the strong association between learning disability and seizures [37]. The higher median co-morbidity scores we noted in the dyskinetic and bilateral spastic type of CP and the severe levels of fine and gross motor function further attests to the fact that the more severe the brain damage the higher the likelihood of the presence of additional impairments [8].

More than two thirds of the cases seen in this study were below the age of five years probably reflecting when care givers seek help for their children at the hospital. The relatively young age may be a result of the clinical manifestations of CP becoming apparent within this time frame. Additionally, children in this age group are very vulnerable to infections and the associated high morbidities and mortalities [38].

There was an apparent decline in the numbers of children with CP in the study with increasing age. The possible reasons for this could be that the caregivers became resigned to the fact that their child with CP would not improve as the child grew older. Alternatively as the child increased in size with age it made it more difficult for the caregivers to bring them to hospital. In addition, rehabilitation facilities for this age group are missing [39], giving little incentive to search for help. Finally, this may be a consequence of decreased survival over time for children with disabilities, which information is uncertain for the sub-Saharan population.

### Limitations

We need to interpret these observations cautiously since our study was conducted at only one institution that is a tertiary care centre which may not be representative of the whole country.

In addition, the cross-sectional design of the study inherently introduces certain limitations in that any causal or directional conclusions cannot be drawn. Furthermore sixteen eligible children were not included due to caregiver issues and these may have affected our results.

The generalizability of our findings is open to discussion because we used the age from 2 years onwards to ascertain our CP-diagnosis and CP subtypes, yet others [6] recommend from the age of 4 years as the clinical subtype may change with time. In addition we did not employ valid and reliably tested psychometric measures of gross and fine motor function.

While advances in diagnostic techniques in HIC have aided the characterization and definition of neurological diseases this is not the case in LMIC [38].

Patients were classified to a single diagnostic category based on their symptoms, examination findings and testing. However, certain conditions could arguably fit into multiple diagnostic categories such as children with severe learning disability by definition are non-verbal, but may be included in the definition of speech and language impairments or children with autistic traits, but do not fulfil an autism spectrum diagnosis.

Finally, a significant amount of information was derived from caregiver interviews using questionnaires. This required good memory and may have been subject to recall bias as well as stating of socially desirable answers.

Despite the study limitations, our findings advance the knowledge of CP in an African setting. This study has the advantage of underscoring the importance of using well-structured history taking and neurological examination as the most useful diagnostic tool as opposed to a chart review where neurological signs and symptoms can easily be missed by a non-neurologist.

## Conclusions

The frequency distribution of CP type and severity is similar to other studies in previous literature in LMIC and HIC however the frequency of the co-morbidities differs with speech and language impairments frequently coexistent. The large proportion of severe bilateral CP, signs of learning disability and epilepsy is possibly caused by birth asphyxia, post-natal infections or varied insults in the last trimester. The majority of these causes is preventable and makes a strong argument for improving maternal and child health care. None the less, since our study is based on a clinical sample it lends support to the call for conducting population based studies on CP in LMIC to determine the prevalence, incidence and other epidemiological features using standard diagnostic tests and CP registers. This calls for concerted efforts from government and stakeholders to rejuvenate the health care systems; and to ensure that adequate funding is provided to advocate for this agenda and commit to improved public and maternal child health.

## Additional files

Additional file 1: Table S1. The Surveillance of Cerebral Palsy in Europe (SCPE) Cerebral Palsy classification. Additional file 2: Table S2. Levels of Gross and Fine Motor Function and Differentiating Criteria. Additional file 3: Appendix S1. Questionnaire for caregiver interview. Additional file 4: Table S3. Clinical type of CP and Level of Fine Motor Function Compared with Development of the Finger Feeding Function. Additional file 5: Table S4. Associations between the co-morbidities and the outcomes of bilateral spastic CP, severe gross motor and fine motor functions.

## Abbreviations

CP: Cerebral palsy
SCPE: Surveillance of cerebral palsy in Europe network
HIC: High income countries
LMIC: Low and middle income countries
IQR: Interquartile range
SSA: sub-Saharan Africa
HIV: Human immunodeficiency virus
HAART: Highly active antiretroviral therapy
