# Supplementary material for: Cerebral palsy in children in Kampala, Uganda: clinical subtypes, motor function and co-morbidities
Source: BMC Res Notes. 2015 Apr 23;8:166. doi: 10.1186/s13104-015-1125-9 (PMC4421914; doi:10.1186/s13104-015-1125-9)
Supplement: Additional file 3: Appendix S1. — Questionnaire for caregiver interview. [file 13104_2015_1125_MOESM3_ESM.pdf]

Cerebral Palsy Study (CPSTUD) ID: I\G|D\0\9___\ ______\ ______

CEREBRAL PALSY STUDY 2009

CLINICAL HISTORY QUESTIONNAIRE

Personal Details

Today's Date:……………………………..………[__]__]/[__]__]/[2_]0_]0_]9_](**TDATE**)

CPSTUD 2009 Number:………..…….[I] G] D] 0] 9 [__]__]__]__]__] (**CPIGD09)**

PID NO:…………………………..………… [ ][ ][ ][ ][ ][ ][ ](**PID**)

Name of child:____ (**NAME**)

Name of home residence:………..…………………………………………… (**RESID**)

DOB: (dd/mm/yyyy)………………………………..[__]__]/[__]__]/[__]__]__]__](**DOB**)
(If not known don't estimate, enter 99/99/9999)

Age: (in completed years & months)……………………………… [__][__].[__][__](**AGE**)
(estimate if date of birth unknown)

Sex: M/F…………………………..…………………...………………..… [__](**SEX**)

Head of Household/ Guardian's Name: ______________________________(**HEADHSE**)

Phone contact: _____________1.[ ][ ][ ][ ][ ][ ][ ][ ][ ][ ](**PHCONT1**)
2.[ ][ ][ ][ ][ ][ ][ ][ ][ ][ ](**PHCONT2**)

Marital Status____ [__] (**MARSTAT**)

1. Married 2.Single 3.Widowed 4.Seperated/Divorced

What is his/her highest level of education ever attained? _____________[__] (**HIGHEDUC**)

1. None
2. P1 – P4
3. P5 – P7
4. S1 – S4
5. S5 – S6
6. Post secondary
7. Tertiary (University).
8. Unknown.

What is his/her major occupation? _______________________________[__] **MAJOCCUP**)

What is his/her main source of income? ___________________________[__] (**MAINCOME**)

Who will answer questions about the index? ………………………………… [__](**QCHILD**)

1. The Index's mother
2. The Index's father
3. Index's aunt
4. Index's uncle
5. Index's grandmother
6. Index's sibling
7. Another relative
8. Other.

Is the informant one who mainly takes care of the Index? (Y/N)…………… [__](**INFORM**)

How many other children stay at home with this child?…………………..[__](**CHILDHME)**

Name of history taker……………………………………………….……..[__](**HISTNAME**)

Date of completion (dd/mm/yyyy)……………………..[__]__]/[__]__]/[__]__]__]__](**DOC**)

Cerebral Palsy Study (CPSTUD) ID: I\G|D\0\9___\ ______\ ______

Child's Medical History

A. **PERCEIVED PROBLEMS**: Ask the parent:

IS THERE ANYTHING ABOUT THE CHILD THAT WORRIES YOU?

Examiner: If yes, inquire about the problem and complete the table below. After recording the information for one problem area, ask about all other problem-areas and complete the table. When no problem is perceived in an area, circle No and leave the remaining box blank for the area. If the answer is no to the first inquiry, still ask specifically about each problem area and complete the table. When more than one option in the table seem to apply, enter the smallest number that is applicable, **except for event associated** enter all that apply.

**PROBLEM AREA**	Does the Parent Perceive a Problem (**Circle** No or Yes	**APPROXIMATE AGE AT ONSET IN MONTHS**<br>At birth=888, D/K=998 (e.g. at 1 month=100) (Estimate if exact age of onset is not known)	**EVENT ASSOCIATED**<br>None=1, Prenatal=2 Birth related=3 LBW=4, Preterm=5 Fever, Infection=6 Injury=7 Malnutrition=8 Other, specify=9	**TREATMENT RECEIVED**<br>None=1 Modern only=2 Folk only=3 Both=4 D/K=8	**FAMILY HISTORY**<br>None=1, Parent=2 Sibling=3 Grandparent=4 1st cousin, aunt, uncle=5 Other blood Relative=6 D/K=8
**Walking**<br>If yes, describe:	No Yes<br>CHQ1	CHQ2	CHQ3	CHQ4	CHQ5
**Hearing**<br>If yes, describe:	No Yes<br>CHQ6	CHQ7	CHQ8	CHQ9	CHQ10
**Vision**<br>If yes, describe:	No Yes<br>CHQ11	CHQ12	CHQ13	CHQ14	CHQ15
**Speech**<br>If yes, describe:	No Yes<br>CHQ16	CHQ17	CHQ18	CHQ19	CHQ20
**Seizures**<br>If yes, describe:	No Yes<br>CHQ21	CHQ22	CHQ23	CHQ24	CHQ25
**Learning**<br>If yes, describe:	No Yes<br>CHQ26	CHQ27	CHQ28	CHQ29	CHQ30
**Behaviour**<br>If yes, describe	No Yes<br>CHQ31	CHQ32	CHQ33	CHQ34	CHQ35
Other:<br>If yes, describe (e.g. other chronic illnesses)	No Yes<br>CHQ36	CHQ37	CHQ38	CHQ39	CHQ40

Additional comments:

Cerebral Palsy Study (CPSTUD) ID: I\G|D\0\9___\ ______\ ______

PREGNANCY HISTORY (For birth of this child):

Gravidity ___ ___ *CHQ41* Stillbirths ___ *CHQ42*

Parity ___ ___ *CHQ43* Spontaneous abortions ___ *CHQ44*

Enter the correct numbers for gravidity, parity.
Gravidity = total number of pregnancies before this child (and counting this child).
Parity = total number of actual births before this child (and counting this child)
Note any stillbirths and spontaneous abortions that applied at the time of this child's birth.

When the mother was pregnant with this child did she have:
Codes: No=1 Yes=2 Don't know=8

Antenatal care ___ *CHQ45* High Blood Pressure ___ *CHQ46*
Bleeding in 1st trimester ___ *CHQ47* Bleeding in 2nd or 3rd trimester ___ *CHQ48*
Diabetes ___ *CHQ49* Infection/Fever in 1st trimester ___ *CHQ50*

Injuries resulting from violence ___ *CHQ51* Poor nutrition ___ *CHQ52*
Treatment with Thyroid hormone ___ *CHQ53*
Treatment with Oestrogen/progesterone hormone ___ *CHQ54*
Treatment with anticonvulsants ___ *CHQ55*
Treatment for a psychiatric disorder___ *CHQ56*
*Any other health problems? ___ *CHQ57*
(If Yes, Specify____)
**(Prompt for signs of UTI .Do not include here problems with veins, moderate vomiting or mild conditions)*

Has the mother *ever* had a goiter? ___ *CHQ58* Has the mother *ever* had epilepsy? ___ *CHQ59*

Age of Father at child's birth___ *CHQ60* Age of the mother at delivery of child (years) ___ *CHQ61*

BIRTH HISTORY:

Where was the child born? ___ *CHQ62*
Codes: Home=1, Hospital=2, Clinic/Maternity Home=3, Other=4, Unknown=8

Was it a single birth? ___ *CHQ63*
Codes: Single birth=1, Twins=2, Triplets or more=3, Unknown=8

Was the baby born at term (between 37-42 weeks or at about 9 months)? ___ *CHQ64*
Codes: Yes=1 No, >3 weeks early=2 No, > 2 weeks late=3, Unknown=8

Was there prolonged rupture of membranes (>24 hours) before delivery? ___ *CHQ65*
Codes: No=1, Yes=2, Don't know=8

How long was the labour? ___ *CHQ66*
Codes: <24 hours=1, >24 hours=2, No labor, C-Section= 3, Unknown=8

Who assisted in delivering the baby? ___ *CHQ67*
Codes: Trained midwife=1, TBA=2, Doctor=3, Family member=4 Other=5 Don't know=8

Was there cord prolapse during labour and/or delivery? ___ *CHQ68*
Codes: No=1, Yes=2, Don't know=8

Was there placenta previa during labour and/or delivery? ___ *CHQ69*
Codes: No=1, Yes=2, Don't know=8

Was there uterine rupture during labour and/or delivery? ___ *CHQ70*
Codes: No=1, Yes=2, Don't know=8

Were there any other difficulties with labour and/or delivery? ___ *CHQ71*
<u>Codes:</u> No=1 Yes=2 (Specify ____) Don't know=8

In what position was the baby born? ___ *CHQ72*
<u>Codes:</u> Head first=1, Feet or buttocks first=2, C-Section=3, Unknown=8

Did the baby cry immediately after birth? ___ *CHQ73*
<u>Codes:</u> Yes=1 No, but in <5 min.=2, No, after>5 minutes=3, Unknown=8

What was the colour of the baby at birth? ___ *CHQ74*
<u>Codes:</u> Normal=1 Blue=2 White=3 Unknown=8

Did the birth attendant have to do anything to the baby to make her/him breathe? ___ *CHQ75*
<u>Codes:</u> No=1 Yes=2 Unknown=8
<u>If yes</u>, describe <u>**why**</u> and <u>**how**</u> breathing was assisted:
____
____
____

Was the baby taken away from the mother immediately after birth? ___ *CHQ76*
<u>Codes:</u> No=1 Yes=2 Unknown=8
If yes, describe circumstances:____
____
____

If the baby was kept in a hospital, for how many days was kept there? ___ ___ *CHQ77*
(Enter 00 if child was not admitted to hospital)

What was the birth weight in kilograms? (9.9=Unknown): ___. ___ *CHQ78*

How big was the baby at birth? ____ *CHQ79*
<u>Codes:</u> About the size of most babies=1, Bigger than most babies=3
Smaller than most babies =2, Don't know=8

Did the child have any difficulties from 28th weeks gestation to the first seven days after birth?
(Perinatal period)
<u>Codes:</u> No=1, Yes=2, Don't know=8

Seizures ____ *CHQ80*
Infection (fever) ____ *CHQ81*
Trouble feeding ____ *CHQ82*
Tetanus ____ *CHQ83*
<u>Deep</u> jaundice ____ *CHQ84*
Diarrhoea ____ *CHQ85*
Difficult breathing ____ *CHQ86*

Did the child have any difficulties from birth to day 28 after birth?
(Neonatal period)
<u>Codes:</u> No=1, Yes=2, Don't know=8

Seizures ____ *CHQ87*
Infection (fever) ____ *CHQ88*
Trouble feeding ____ *CHQ89*
Tetanus ____ *CHQ90*
<u>Deep</u> jaundice ____ *CHQ91*
Diarrhoea ____ *CHQ92*
Difficult breathing ____ *CHQ93*

Did the child have any of these conditions from one month of age to the age of 2 years? (**Post neonatal)**
Codes: No=1, Yes=2, Don't know=8

Seizures _____ *CHQ94*
Febrile convulsions _____ *CHQ95*
Meningitis ____ *CHQ96*
Tetanus _____ *CHQ97*
Deep jaundice _____ *CHQ98*
Cerebral Malaria _____ *CHQ99*
Difficult breathing _____ *CHQ100*
Trauma to head _____ *CHQ101*
Hydrocephalus _____ *CHQ102*

EPILEPSY HISTORY:

Has child ever suffered from epilepsy or multiple seizures? ______ *CHQ103*
(i.e. Two or more unprovoked seizures excluding neonatal & febrile seizures) Codes: No=1, Yes=2, Don't know=8

If yes, verbal account ____

____

____

If yes, is child on medication for seizures? ______ *CHQ104*
Codes: No=1, Yes=2, Don't know=8, (Enter 0 if child has no seizures)

Which drugs is child on? ______ ______ ***(List name(s))*** *CHQ105*
******(Enter 00 if child has no seizures)********

FAMILY HISTORY:

Are the parents of the child related to each other by blood? ______ *CHQ106*
No=1, Yes, as first cousins=3 Yes, as distant cousins=5
Yes, as uncle and niece=2 Yes, as second cousins=4 Don't know=8

Is there any other member of the family with a similar condition? ______ *CHQ107*
Codes: No=1 Yes=2 (Specify who____) Don't know=8

NUTRITIONAL HISTORY:

Was the child breast-fed and for how long? _____ *CHQ108*
Codes: No, never=1 Yes, 1-6 mo=3 Yes, 13-18 m=5 Yes, >24 mo=7
Yes, <1 mo=2 Yes, 7-12 mo=4 Yes, 19-24 mo=6 Unknown =8

When did the child first start other milks other than breast milk? _____ *CHQ109*
Codes: Never=1 1-6 mo=3 13-18 mo=5 After 24 mo=7
<1 mo=2 7-12 mo=4 19-24 mo=6 Unknown=8

At what age in months was solid food introduced? _____ _____ *CHQ110*
(Enter 77 if not yet, 88 if unknown)

What are the three main types of foods that child is fed on?(998=Unknown) _____ _____ _____ *CHQ111*
Codes: Legumes (Beans, G/Nuts/Peas) =1 Posho/Millet or Cassava bread=4 Milk =7
Fish=2 Matooke/Potatoes/Cassava=5 Porridge =8
Meat =3 Rice= 6 Green vegetables=9

How often is child fed with above food within a 24 hour period? _____ *CHQ112*
Codes: Once=1 Twice=2 Thrice=3 Four times=4 More than 4X/day=5 Unknown=8

What is the consistency of the feeds? _____ *CHQ113*
Codes: Liquids=1 Semi-solids=2 Solids=3 Both Solids and Liquids=4

Can the child feed himself or herself? ____ *CHQ114*
(assess in accordance to local cultural norms) Codes: Yes, skillfully (with spoon/fork or fingers)=1
No, must be fed=3,Yes, but unskilled (i.e. like a baby)=2, Unknown=8

Does child have any feeding problems? ____ *CHQ115*
Codes: No=1, Yes=2, Don't know=8

If child has a feeding problem, which one of these is the most dominant one? ____ *CHQ116*
(Enter 0 if no feeding problem)
Swallowing difficulties=1 Regurgitation of feeds=3 Tongue thrust=5 Tonic bite=7
Chewing difficulties=2 Vomiting of feeds=4 Drooling saliva=6

DEVELOPMENTAL HISTORY:

At what age did child attain the first social smile? ____ *CHQ117*
Codes: By 6wks=1, By 4mo =2, By 6mo=3, By 8mo=4, By 1 year=5, Not yet=6 Unknown=8

At what age did child sit without support? ____ *CHQ118*
Codes: By 9mo=1, After 12mo but before 18mo=2, After 18mo but before 24mo= 3,
After 24mo but before 30 mo=4, After 30mo but before 36mo=5, Not yet=4, Unknown=8

At what age did the child walk without help or holding on? ____ *CHQ119*
Codes: By 18 mo=1 By 2 years=2 After 2 years=3 Not yet=4 Unknown=8

At what age did child start to finger feed himself or herself? ____ *CHQ120*
Codes: By 7mo=1, By 1 year=2, After 1 year=3 After 2 years=4, Not yet=5 Unknown=8

At what age did child start speaking two word sentences? ____ *CHQ121*
Codes: By 24mo=1, By 30 mo=2, After 30mo=3, After 36mo=4, Not yet=5, Unknown=8

SPEECH AND LANGUAGE FUNCTIONS:

Child's mode of communication________ *CHQ122*
Codes: None=1, Nonverbal=2, Verbal=3, Verbal and Nonverbal=4

Child's verbal expression________ *CHQ123*
Codes: None/ only crying=1, Vocalization=2, Words=3, Phrases=4, Sentences=5

Child's Verbal Comprehension________ *CHQ124*
Codes: None=1, Words=2, Phrases=3, Simple sentences=4, Complex sentences=5

Child's Non verbal Expression________ *CHQ125*
Codes: None=1, Poor=2,Gestures=3, Signs=4

Child's Nonverbal Comprehension________ *CHQ126*
Codes: None=1,Poor=2,Gestures=3, Facial expressions=4, Signs=5

Child's Intelligibility of speech________ *CHQ127*
Codes: None=1,Poor=2, Fair=3,Good=4

AUTISM

Ask the mother/ main caregiver the following questions:
Codes: No=1 Sometimes=2 Most of the times=3

Does the child **point** to indicate **something interesting**? ________ *CHQ128*
Does the child commonly **say the same thing** over and over in almost exactly the same way?
________ *CHQ129*
Does the child have any unusual **repetitive movements**? (like finger tapping, hand flapping, head banging, object banging, circling) ________ *CHQ130*

Does the child appear to be **lost in his/ her own world**? (Alinga ali aloota "Dreamworld" - use local terms) ________ *CHQ131*
Does the child **respond to his/ her name**? ________ *CHQ132*
Does the child **engage in pretend play?** (like Mummy& Daddy, Doctor, Teacher, Shopkeeper) ________ *CHQ133*
Does the child **make gestures?** (like Come or Bye-Bye) ________ *CHQ134*
Does the child **often bring objects over to you** (caregiver/parent) to show you something? ________ *CHQ135*
Does the child **insist** upon **a particular routine**? (local examples: insistence on same food habits, wearing same dress/shirt, same cup/plate) ________ *CHQ136*
Does the child have **difficulty in** making and maintaining **eye contact**? ________ *CHQ137*

Observation of Function: Complete for all children.
Instructions: Observe the child carry out **7 tasks** listed below:

1. Observe the child walking at least 5 steps into room. Watch carefully, looking for limp, asymmetry of gait, toe walking, ataxia, involuntary movement, and atrophy or contracture.

2. Welcome the child and observe the response: Does he or she hear, make an appropriate social response, smile, act shy, or speak?

3. Invite the child to squat and pick up a tiny object, such as a bead, coin or raisin (defined size) using each hand in turn. Observe carefully for fisting, asymmetry in grasp, absence of pincer grasp or difficulty in seeing the object.

4. Observe the child as he/she stands up: Does he/she need to use hands to get to an upright position? (proximal muscle weakness)

5. Elicit speech by asking the child questions such as: "What did you pick up? " What is that?" (point to a raisin, chair etc.) "What is this called?" (point to nose, ear, tooth etc.) "What is your name?" Watch for problems in hearing, speech and comprehension.

6. Ask the child to point to body parts (eyes, mouth etc.) Observe for problems in hearing and comprehension.

7. Give the child paper and a pencil and ask him or her to draw something. Scribble (for 2 year old) or draw shapes: circle (for 3 year old), square (for 4 through 6 years' old), diamond (for 7 through 9 years' old). Observe the motor function and comprehension.

Rate the child in the following areas after observing the above 7 tasks:

Codes: Pass=1 Fail=2 Uncertain=3
Gross motor ____ *CHQ138*
Hearing ____ *CHQ139*
Vision ____ *CHQ140*
Speech (motor) ____ *CHQ141*
Speech (language) ____ *CHQ142*
Comprehension ____ *CHQ143*
Fine motor ____ *CHQ144*

Physician/Nurse/Research Assistant: Codes: No=1 Yes=2 Uncertain=8.
Do you think, based on the interview with the informant and this brief observation that the child has autistic features? ____ *CHQ145*

Do you think, based on the interview with the informant that child has epilepsy? ____ *CHQ146*

Sign if you have checked that the form is complete____________________________
